# Présentations de l'adénite tuberculeuse de la tête et du cou au CHU de Bobo-Dioulasso, Burkina Faso

**DOI:** 10.11604/pamj.2013.15.131.2919

**Published:** 2013-08-10

**Authors:** Rasmané Béogo, Noraogo Emile Birba, Toua Antoine Coulibaly, Ibraïma Traoré, Kampadilemba Ouoba

**Affiliations:** 1 Service de stomatologie et de chirurgie-maxillo maciale, CHU Sanou Souro, Bobo-Dioulasso, Burkina Faso; 2 Service d'ORL et de chirurgie cervico-faciale, CHU Yalgado Ouédraogo, Ouagadougou, Burkina Faso

**Keywords:** Adénite, Tête et cou, Tuberculose, VIH, adenitis, head and neck, Tuberculosis, HIV

## Abstract

Les ganglions de la tête et du cou sont parmi les localisations les plus fréquentes de la tuberculose, un problème de santé publique dans le monde. Une étude rétrospective conduite entre 2001 et 2010 rapporte les caractéristiques épidémiologiques et cliniques de l'adénite tuberculeuse de la tête et du cou, au CHU Sanou Souro, au Burkina Faso. Au total, 115 patients ont été observés dont l'âge était compris entre 2 ans et 64 ans (moyenne 31,46 ans). Il y avait 53 patients de sexe masculin (46,1%) et 62 de sexe féminin (53,9%). Un pic de fréquence de 39,8% était observé entre 30 et 39 ans. Les adénopathies cervicales étaient multiples chez 96,5% des patients et abcédées chez 30%. Elles étaient associées à des adénopathies extra cervicales chez 16,6% des patients. Chez 83,4% des patients, il a été noté un ou plusieurs signes à type d'asthénie et ou d'amaigrissement (70,8%), de fièvre 25% ou de toux (20,8%). L'infection associée la plus fréquente était celle par le VIH, observée chez 43,3% des patients. Les résultats de cette étude commandent la recherche systématique de l'infection par le VIH chez tout patient porteur d'adénite cervicale tuberculeuse dans un contexte de double endémicité de la tuberculose et de l'infection à VIH.

## Introduction

La tuberculose est un problème de santé publique, affectant un tiers de la population mondiale et représentant la quatrième cause de décès [1]. La tuberculose ganglionnaire est la forme de tuberculose extra pulmonaire la plus fréquente dans les zones à faible prévalence de l'endémie tuberculeuse [2]. Dans les zones à forte prévalence de l'endémie dont le Burkina Faso, elle occupe le deuxième rang après la pleurésie tuberculeuse [2]. L'adénite tuberculeuse est la forme clinique la plus fréquente de la tuberculose de la tête et du cou [1, 3]. A l'instar des autres formes de la tuberculose extra pulmonaire, la tuberculose ganglionnaire de la tête et du cou peut être associée à une localisation pulmonaire et à l'infection à VIH [4].

Le but de cette étude était de décrire l'épidémiologie et les caractéristiques cliniques de l'adénite tuberculeuse de la tête et du cou, au CHU Sanou Souro, au Burkina Faso.

## Méthodes

Nous avons conduit une étude rétrospective et descriptive sur dix ans, entre 2001 et 2010, dans les services de stomatologie et chirurgie maxillo faciale et de phtisiologie du CHU Sanou Souro de Bobo-Dioulasso. Tous les patients ont bénéficié d'un examen clinique, d'une numération formule sanguine et d'une radiographie du thorax. La sérologie de l'infection par le VIH était en outre pratiquée quand le patient y consentait. Le diagnostic de la tuberculose ganglionnaire était fait sur un ou plusieurs des critères suivants: la mise en évidence de granulome tuberculoïde et de nécrose caséeuse à l'examen histologique d'une pièce de biopsie ganglionnaire; la mise en évidence de bacilles acido alcoolo résistants (BAAR) à l'examen bactériologique du produit de biopsie ganglionnaire. Les variables étudiées étaient les caractéristiques démographiques du patient (âge et sexe), les caractéristiques cliniques de l'adénopathie (nombre, consistance, siège, signes associés, pathologies associées). Le test de Chi2 a été utilisé pour la comparaison des variables qualitatives; la différence était significative quand p<0,05.

## Résultats

### Caractéristiques des patients

**Figure 1:**
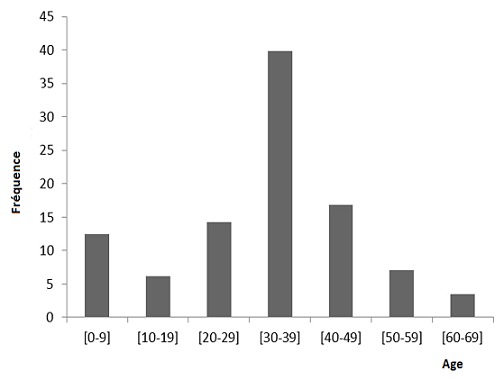
Distribution des patients selon l'âge

Sur une période de 10 ans, au total 115 patients étaient porteurs d'adénites tuberculeuses, soit une fréquence annuelle de 11,5 patients. L'âge des patients était compris entre 2 ans et 64 ans; l'âge moyen était de 31,46 ans. Un pic de fréquence de 39,8% était observé entre 30 et 39 ans (Figure 1). Dix neuf patients (16,5%) avaient moins de 16 ans. Il y avait 62 patients de sexe féminin (53,9%) et 53 patients de sexe masculin (46,1%) soit un rapport femme/homme de 1,2.

### Caractéristiques cliniques des adénopathies

**Figure 2:**
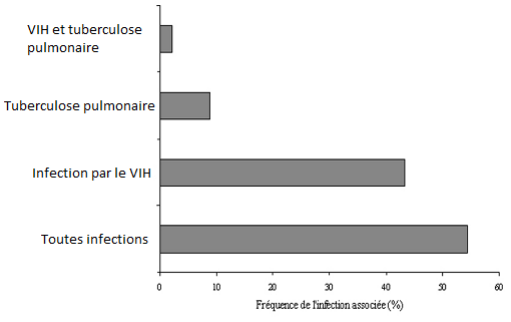
Fréquence des infections associées à l'adénite tuberculeuse

Chez 111 patients (96,5%), les adénopathies cervicales étaient multiples. Chez 19 patients (16,6%), la localisation cervicale était associées à des localisations axillaires (11 patients), médiastinales (7 patients) ou inguinales (4 patients). Chez 34 patients (30%), des adénopathies fluctuantes ou fistulisées étaient observées, à la consultation. Chez 96 patients (83,4%), il a été noté un ou plusieurs signes associés. Une altération de l'état général à type d'asthénie et ou d'amaigrissement était le signe le plus fréquent, observé chez 81 patients (70,4%). La fièvre était observée chez 29 patients (25,2%) et la toux l'était chez 24 patients (20,8%). Chez 54,4% des patients (49/90), une ou plusieurs infections étaient associées à la tuberculose ganglionnaire au premier desquelles, l'infection par le VIH, observée chez 43,3% des patients (39/90) (Figure 2).

## Discussion

La tuberculose est l'étiologie la plus fréquente des adénopathies chroniques de la tête et du cou dans les pays en développement [5, 6]. L'adénite tuberculeuse est une affection de tous les âges [5, 7] comme le montrent les âges extrêmes des patients dans cette étude. Sa prédilection dans cette étude pour des sujets jeunes est conforme à l'épidémiologie de la tuberculose dans les pays en développement à l'instar du nôtre. En Inde, des études rapportent une moyenne d'âge des patients de 23,7 ans et un pic de fréquence de l'affection entre 11 et 30 ans [5, 8]. Aux USA et au Royaume Uni, ce pic est rapporté entre 25 et 50 ans [9, 10]. L'exposition au bacille tuberculeux est plus précoce et plus forte dans les pays en développement en raison de la forte prévalence de la tuberculose et de la précarité des conditions d'hygiène [2]. La légère prédominance féminine avec un rapport femme/homme de 1,2 observée dans cette étude est aussi rapportée par d'autres auteurs [5, 11]. L'adénite tuberculeuse cervicale peut être associée à une diversité de signes généraux. Parmi ces signes, l'asthénie, l'amaigrissement et la fièvre, observés chez la plupart des patients dans cette étude, sont rapportés à des fréquences variables par certains auteurs. La fièvre et l'amaigrissement sont observés respectivement chez 28% et 12% des patients par Prasad et al [4]. L'asthénie et l'amaigrissement sont rapportés aux fréquences respectives de 18% et 14% par Jha et al. [5]. Dandapat et al rapportent une perte de poids et la fièvre à des fréquences de 85% et 40% [11]. L'asthénie ou l'amaigrissement observés chez 70,8% dans cette étude peuvent s'expliquer par une longue évolution de l'adénite tuberculeuse et ou la présence de pathologies associées, en particulier la tuberculose pulmonaire et l'infection à VIH. La présence des signes généraux corrobore la théorie qui dit de la tuberculose ganglionnaire, la manifestation locale d'une infection systémique [12]. Le bacille tuberculeux atteint le système lymphatique à partir d'un foyer primaire du poumon ou de l'anneau de Waldeyer [13, 14]. Plus de 90% des personnes infectées guérissent de cette primo infection grâce au système immunitaire [2]. L'adénite tuberculeuse se développe après la réactivation de bacilles quiescents des ganglions le plus souvent ou plus rarement, après une exposition directe à l'infection [15]. L'observation d'adénites abcédées, à la consultation, chez 30% des patients, est un témoin du faible accès aux services de santé dans les pays à faibles à ressources limitées. La présence d'adénites cervicales multiples observée chez la plupart des patients est classique [7, 16, 17]. Elle constitue selon Desa, un critère d'orientation diagnostique de l'adénite tuberculeuse [18]. La tuberculose est en outre classiquement décrite parmi les causes d'adénopathies généralisées [5]. A l'adénite cervicale, peuvent s'associer des adénopathies axillaires, inguinales, médiastinales ou péritonéales. La prépondérance de l'adénite cervicale isolée observée dans cette étude est rapportée par certains auteurs [5, 11]. La fréquence de l'association entre la tuberculose et l'infection à VIH est rapportée par de nombreux auteurs. Le déficit immunitaire augmente aussi bien le risque de développement d'une infection tuberculeuse récente que celui de la réactivation d'une infection quiescente [19]. Prasad rapporte l'infection par le VIH chez 30% des patients avec une tuberculose de la tête et du cou [4]. Kamana rapporte la tuberculose comme la première étiologie d'adénopathies chez les personnes infectées par le VIH en Inde [19]. La tuberculose pulmonaire est aussi l'une des pathologies associées les plus fréquentes de la tuberculose ganglionnaire dont elle peut être le foyer de départ. Priel et al rapportent la tuberculose pulmonaire chez 28,8% des patients avec une lymphadénopathie tuberculeuse [20]. Prasad et al. rapportent une fréquence de la tuberculose pulmonaire chez 24, 2% des patients avec une tuberculeuse de la région de la tête et du cou [4].

## Conclusion

Dans cette étude, l'adénite tuberculeuse se caractérise par des adénopathies multiples, parfois abcédées, souvent associées à des signes généraux, chez un sujet qui est volontiers un adulte jeune. Son association fréquente à l'infection à VIH indique la recherche systématique de cette infection chez tout sujet avec une adénite tuberculeuse.
